# Development of Novel PET Tracers for Imaging Non-AD Tauopathies

**DOI:** 10.1021/acsmedchemlett.5c00487

**Published:** 2025-09-03

**Authors:** Steven H. Liang

**Affiliations:** Department of Radiology and Imaging Sciences, 1371 Emory University, 1364 Clifton Road, Atlanta, Georgia 30322, United States

**Keywords:** Tauopathy, positron emission tomography, neurodegenerative disease, non-Alzheimer’s disease tauopathies, in vivo imaging

## Abstract

Tauopathies are a heterogeneous group of neurodegenerative diseases characterized by the pathological accumulation of microtubule-associated protein tau filaments in the brain. The development of tau-targeted positron emission tomography (PET) tracers has facilitated in vivo mapping and quantification of this pathological biomarker, particularly in Alzheimer’s disease (AD). Recent efforts have expanded toward the development of radioligands for imaging non-AD tauopathies, which are critical for advancing diagnosis and understanding disease mechanisms across the broader spectrum of tau-related neurodegeneration.

## Graphical abstract

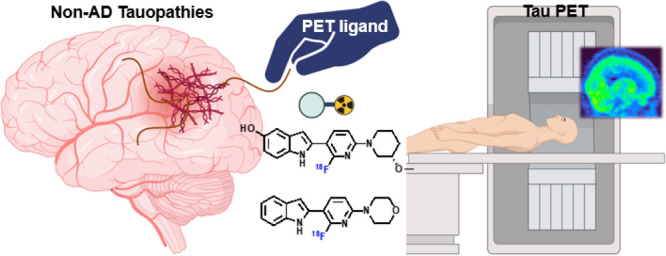

Tau is encoded by a single gene named microtubule associated protein tau (*MAPT*) and located on human chromosome 17q21.31.^1^ Tau is predominately expressed in the central and peripheral nervous systems with the highest concentrations found in neuronal axons. It promotes microtubule polymerization, assembly, and stabilization, thereby facilitating axonal transport and maintaining neuronal structural integrity.^2–4^ In the adult human brain, six different tau isoforms are produced through alternative splicing of exons 2, 3, and 10 of *MAPT* mRNA. As illustrated in Figure 1, alternative splicing of exons 2 and 3 generates isoforms classified by *N*-terminal inserts as 0N (both exons excluded), 1N (only exon 2 included), or 2N (both exons included). Splicing at exon 10 determines the number of microtubule-binding (MTB) repeats, yielding either three-repeat (3R) isoforms (lacking exon 10, containing repeats R1/R3/R4) or four-repeat (4R) isoforms (containing exon 10, with repeats R1–R4). These combinations defined six distinct tau isoforms (0N3R, 0N4R, 1N3R, 1N4R, 2N3R, 2N4R) that range from 352 to 441 amino acids in length.^2,3^

**Figure 1:**
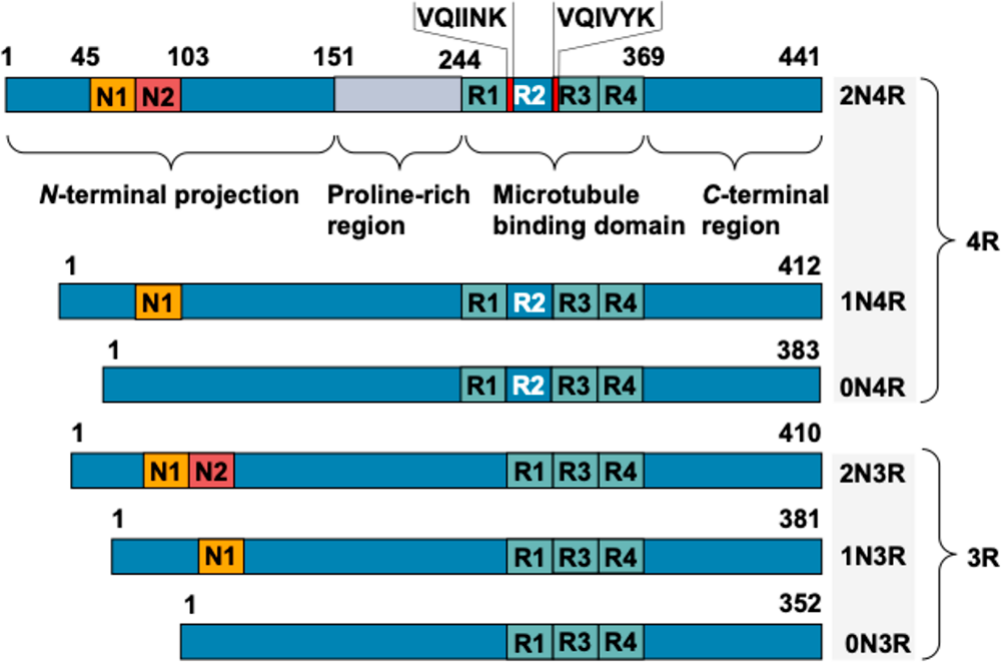
Schematic structure of tau isoforms. The longest isoform of human tau present in the brain is shown with four regions: *N*-terminal projection domain (encoded by exon 2 and 3), proline-rich region, microtubule binding (MTB) repeats (R1–R4, R2 is encoded by exon 10), and *C*-terminal region. The hexapeptide sequences VQIINK and VQIVYK are indicated in red.

As depicted in Figure 1, tau comprises four structural domains: a *C*-terminal domain, an MTB repeat domain, a proline-rich region, and an *N*-terminal projection domain. Located within the C-terminal region, the 3R or 4R repeats span residues 244–368 in 2N4R and 252–376 in 2N3R isoforms, respectively.^3^ Two hexapeptide sequences VQIINK at the beginning of R2 and VQIVYK at the beginning of R3 are essential for tau aggregation.^2,3^ In the healthy adult cortex, 3R and 4R tau isoforms are expressed at approximately equal levels, whereas a higher level of 3R tau is found in early brain development.^5^ Under pathological conditions, tau transitions from a soluble, intrinsically disordered protein to an insoluble, fibrillar conformation with a cross-β structure. This aggregation process is primarily driven by post-translational modifications (PTMs), predominantly hyperphosphorylation, along with acetylation and ubiquitination.^2,3,6^ As illustrated in Figure 2A, tau hyperphosphorylation disrupts tau-microtubule interactions, causing microtubule destabilization and tau self-aggregation and fibrillization. Subsequent oligomerization and fibrillogenesis yield insoluble paired helical filaments (PHFs), which further assemble into tau aggregates. These prion-like aggregates seed soluble tau assembly, propagating pathology that ultimately causes neuronal death and brain damage.

**Figure 2:**
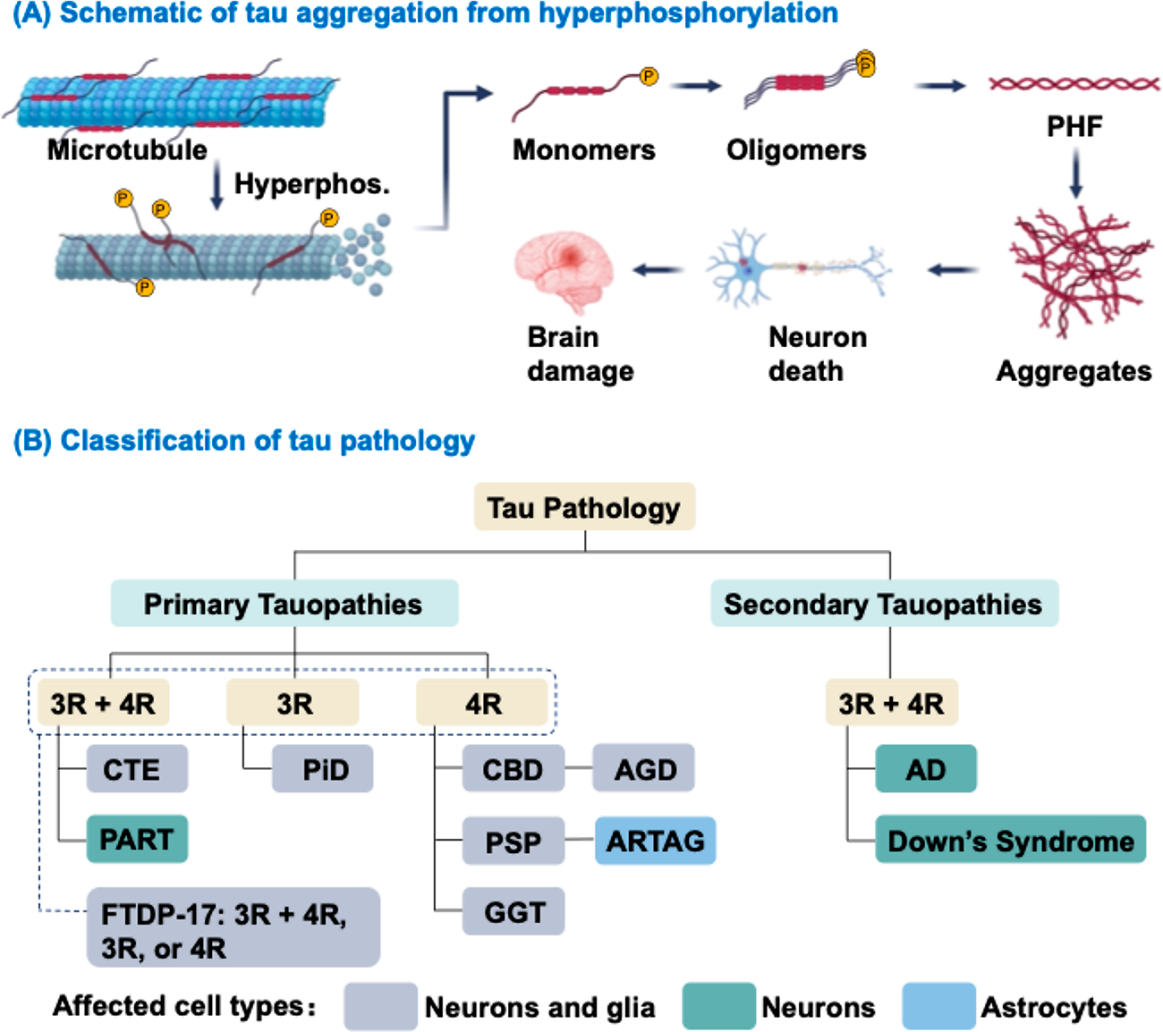
(A) Schematic of tau aggregation that induced by hyperphosphorylation. The self-assembly of soluble tau monomers gives rise to oligomers and PHF, which then results in insoluble aggregates. These aggregates form intracellular inclusions that cause neuronal death and subsequent brain damage. (B) The classification of tauopathies based on the presence of the predominant pathological tau isoforms. In primary tauopathies, tau pathology dominates; in secondary forms, it occurs alongside other protein aberrations. These neurodegenerative disorders affected different brain cells including neurons, glia, and astrocytes. AD, Alzheimer’s disease; AGD, argyrophilic grain disease; ARTAG, aging-related Tau astrogliopathy; CBD, corticobasal degeneration; CTE, chronic traumatic encephalopathy; GGT, globular glial tauopathy; Hyperphos., hyperphosphorylation; PART, primary age-related tauopathy; PHF, paired helical filament; PiD, Pick’s disease; PSP, progressive supranuclear palsy. Reproduced with permission from ref 4. Copyright 2025 American Chemical Society.

Tauopathies constitute a class of neurodegenerative disorders defined by abnormal tau filamentous inclusions within brain cells.^2–4^ These inclusions predominantly affect neurons, glial cells (including astrocytes), with rare occurrence in the extracellular space. Pathological tau forms intracellular inclusions within brain cells, which serve as key biomarkers for various neurodegenerative disorders. The morphology of these inclusions varies by disease subtype and depends on the specific tauopathy, such as neurofibrillary tangles (NFTs), Pick bodies, tufted astrocytes, astrocytic plaques, globular glial inclusions, and coiled bodies. Tauopathies are classified as primary or secondary based on aggregate composition, and further categorized by dominant tau isoform as 3R, 4R, or mixed 3R/4R tauopathies (Figure 2B). In primary tauopathies (Figure 2B, left panel), tau is the major and prominent component of the pathology, as seen in chronic traumatic encephalopathy (CTE, mixed 3R/4R), Pick’s disease (PiD, 3R), and corticobasal degeneration (CBD, 4R). In addition, frontotemporal dementia with parkinsonism linked to chromosome 17 (FTDP-17) features abundant filamentous tau inclusions of 3R, 4R, and mixed 3R/4R. In contrast, secondary tauopathies (Figure 2B, right panel) such as Alzheimer’s disease (AD) and Down syndrome-related dementia also involve tau aggregates, typically mixed 3R/4R isoforms, but occur alongside other primary pathologies, notably amyloid-β deposition.

## Development of Tau PET Radioligands

Positron emission tomography (PET) imaging is widely employed for in vivo visualization and quantification of diverse pathologies,^7–9^ including cancers and neurodegenerative disorders.^10–17^ Neurofibrillary tangles (NFTs), a hallmark of AD, were first identified in 1906.^3^ The development of tau-specific PET tracers has substantially advanced AD research. Several tau PET tracers, exemplified by [^18^F]­flortaucipir ([^18^F]­T807, Tauvid, approved by the U.S. FDA in 2020) and [^18^F]­MK-6240, have proven effective in imaging AD-associated tau aggregates, which compose predominately of mixed 3R/4R isoforms.^4,6^ However, for non-AD tauopathies, these tracers exhibit suboptimal sensitivity and specificity.^4,18–20^ Alternative efforts have included the use of [^18^F]­PI-2620 ^21–23^ and its structural analog [^18^F]­F-4,^24^ as well as [^18^F]­APN-1607 (also known as [^18^F]­PM-PBB3).^25^ Both [^18^F]­PI-2620 and [^18^F]­APN-1607 have shown potential in detecting non-AD tauopathies in PSP,^4^ and have recently received the FDA Fast Track Designation. In addition, further validation is required to establish [^18^F]­F-4 as a reliable PET tracer for non-AD tauopathies. Thus, there is an urgent need to develop PET ligands for non-AD tauopathies, though complicated by the heterogeneity of tau filament structures and low abundance of across diseases, such as 3R and 4R tau in PiD and PSP, respectively.

Despite these challenges, significant progress has been made in the development of indole-based tau PET ligands. [^18^F]­CBD-2115 (also known as [^18^F]­OXD-2115 or [^18^F]**1**), a first-in-class 4R-tau tracer, exhibited limited BBB penetration in rodents and nonhuman primates (NHPs), with standardized uptake values (SUVs) of 0.5–0.65. In vitro autoradiography (ARG) with [^3^H]**1** demonstrated moderate to high affinity for tau aggregates in post-mortem tissues of PSP (*K*_D_ = 4.9 nM) and CBD (*K*_D_ = 27 nM), both dominated by 4R-tau.^26^ However, [^3^H]**1** also bound AD tau aggregates (*K*_D_ = 5.5 nM) in the same binding pockets as [^3^H]­MK-6240 and [^3^H]­flortaucipir, indicating its limited selectivity to 3R/4R tau. Subsequent chemical fingerprint searches led to the discovery of compounds **2** and **3**, which possessed a pyrimidine moiety and showed a shift in binding site preference toward the PM-PBB3 binding site on tau aggregates in AD, PSP, and CBD brain tissues.^27^ Furthermore, [^3^H]**2** (*K*_D_ < 5.5 nM) and [^3^H]**3** (*K*_D_ < 2 nM) exhibited high binding affinity in AD, PSP, and CBD tissues, but lower for α-synuclein (>80 nM) and Aβ. PET imaging in rats with [^18^F]**2** and [^18^F]**3** demonstrated good brain penetration (1.7 vs. 2.8 SUV within 5 min) and rapid clearance (0.4 vs. 0.5 SUV at 120 min) from normal brain tissues, with [^18^F]**3** exhibiting higher brain penetration and faster clearance than [^18^F]**2**. [^18^F]**4** emerged from structure–activity relationship studies, which displayed high affinity for AD, PSP, CBD, and PiD (3.8 nM) tau tissues (*K*_D_ < 4 nM, Figure 3A).^28^ In addition, weak binding in control, Parkinson’s disease (PD), and TDP-43 tissues was observed for [^18^F]**4**, suggesting minimal off-target binding. ARG with [^3^H]**4** revealed specific binding in AD, PSP, and CBD tissues (Figure 3B), which were colocalized with phospho-tau but not Aβ plaques. No specific binding occurred in elderly control tissues (CT, Figure 3B). As shown in Figure 3C, NHP PET imaging of [^18^F]**4** revealed high peak SUVs (∼4–5 SUV) and rapid nonspecific binding clearance (cleared by 3.9-fold in the whole brain within 90 min), indicating favorable properties for clinical translation. Building on the chemical scaffold of **1**, [^18^F]­OXD-2314 ([^18^F]**5**) was developed as a high-affinity radiotracer for various human tau aggregates. It demonstrated strong binding affinity for tau fibrils in AD (*K*_D_ 3.6 nM), PSP (2.3–2.4 nM), CBD (2.1–2.2 nM), and PiD (1.1 nM), while exhibiting markedly reduced binding for α-synuclein (62 nM).^29^ In vitro ARG confirmed the specific and displaceable binding of [^18^F]**5** in AD, CBD, and PSP brain tissues that aligned with phospho-tau (AT8) immunohistochemistry, and minimal binding signal in healthy controls. Preclinical PET imaging in rats and NHPs demonstrated favorable brain uptake and rapid clearance with low radiometabolite levels in the brain. Furthermore, [^18^F]**5** has been successfully translated to first-in-human studies in healthy volunteers.^30^ Dynamic PET imaging indicated rapid brain uptake (2.1 g/mL at ∼2 min postinjection) and fast washout, with uniform distribution across brain regions. Kinetic modeling supported a reversible binding profile. Ongoing clinical studies are currently characterizing the performance of [^18^F]**5** in patients with non-AD tauopathies.

**Figure 3:**
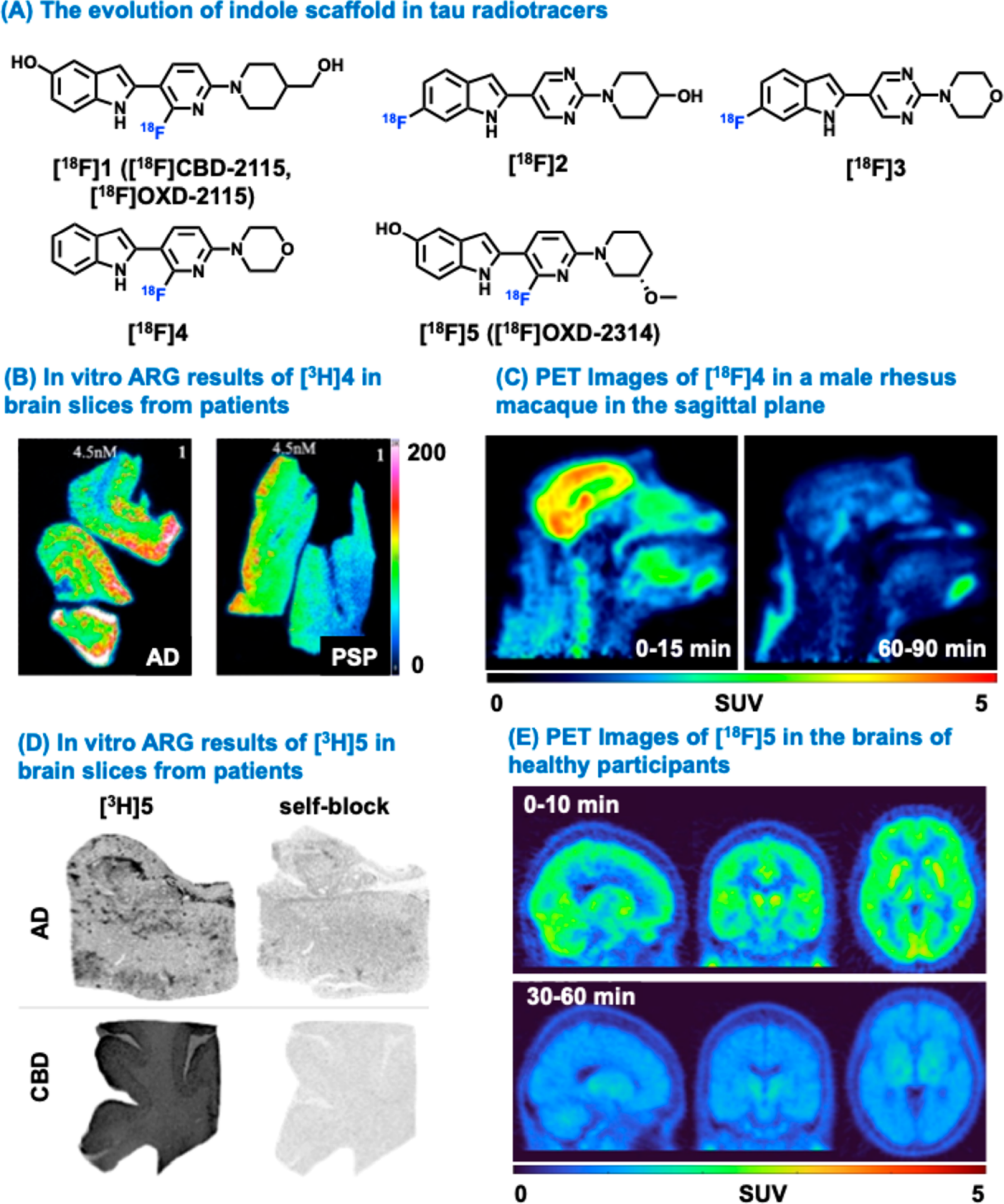
Indole derivatives for PET imaging of tauopathies. (A) Chemical structures of indole-derived tau PET ligands ([^18^F]**1**–**5**). (B) Representative in vitro autoradiography (ARG) results of [^3^H]**4** (4.5 nM) in brain sections from patients of AD and PSP. (C) Summed PET images (0–15 min and 60–90 min) of [^18^F]**4** in a male rhesus macaque. (D) Representative in vitro ARG results of [^3^H]**5** in brain sections from patients of AD and CBD under conditions of both baseline and self-block. (E) Representative PET images (0–10 min and 30–60 min) of [^18^F]**5** in a healthy subject. AD, Alzheimer’s disease; CBD, corticobasal degeneration; PSP, progressive supranuclear palsy. Panels B, C, D, and E were reproduced with permission from ref 28 (Copyright 2025 American Chemical Society)^29^, (Copyright 2025 Springer Nature), and^30^ (Copyright 2025 Springer Nature), respectively.

## Future Outlook

The development of tauopathy-specific PET ligands represents a pivotal frontier in neuroimaging research, with the potential to significantly improve the accuracy of diagnosis, disease staging, and the evaluation of therapeutic efficacy for tau-related neurodegenerative disorders. Although current tracers, such as [^18^F]­flortaucipir and [^18^F]­MK-6240, demonstrate strong binding to mixed 3R/4R tau aggregates in AD, their limited tau subtype selectivity renders them suboptimal for imaging non-AD tauopathies. A deeper structural understanding of the distinct folding patterns and morphologies of tau filaments across different tauopathies is crucial for the rational design of highly selective ligands capable of distinguishing between 3R and 4R tau isoforms. Recent advances in indole derivatives highlight the potential of this scaffold for broadening the scope of PET imaging to encompass a wider spectrum of tauopathies.

## Notes

No unexpected or unusually high safety hazards were encountered

## Abbreviations

PET: positron emission tomography
AD: Alzheimer’s disease
MAPT: microtubule associated protein tau
MTB: microtubule-binding
3R: three-repeat
4R: four-repeat
PTMs: post-translational modifications
PHFs: paired helical filaments
AGD: argyrophilic grain disease
ARTAG: aging-related Tau astrogliopathy
CBD: corticobasal degeneration
CTE: chronic traumatic encephalopathy
GGT: globular glial tauopathy
PART: primary age-related tauopathy
PiD: Pick’s disease
PSP: progressive supranuclear palsy
NFTs: neurofibrillary tangles
FTDP-17: frontotemporal dementia with parkinsonism linked to chromosome 17
NHPs: nonhuman primates
ARG: autoradiography
CT: control tissues

## References

[1] Neve R. L., Harris P., Kosik K. S., Kurnit D. M., Donlon T. A. (1986). Identification
of cDNA clones for the human microtubule-associated protein tau and
chromosomal localization of the genes for tau and microtubule-associated
protein 2. Brain Res..

[2] Gotz J., Halliday G., Nisbet R. M. (2019). Molecular
Pathogenesis of the Tauopathies. Annu. Rev.
Pathol..

[3] Goedert M., Eisenberg D. S., Crowther R. A. (2017). Propagation of Tau Aggregates and
Neurodegeneration. Annu. Rev. Neurosci..

[4] Wongso H., Harada R., Furumoto S. (2025). Current Progress
and Future Directions
in Non-Alzheimer’s Disease Tau PET Tracers. ACS Chem. Neurosci..

[5] Lacovich V., Espindola S. L., Alloatti M., Pozo Devoto V., Cromberg L. E., Čarná M.
E., Forte G., Gallo J.-M., Bruno L., Stokin G. B., Avale M. E., Falzone T. L. (2017). Tau Isoforms Imbalance Impairs the Axonal Transport
of the Amyloid Precursor Protein in Human Neurons. J. Neurosci..

[6] Sexton C., Snyder H., Beher D., Boxer A. L., Brannelly P., Brion J. P., Buee L., Cacace A. M., Chetelat G., Citron M., DeVos S. L., Diaz K., Feldman H. H., Frost B., Goate A. M., Gold M., Hyman B., Johnson K., Karch C. M., Kerwin D. R., Koroshetz W. J., Litvan I., Morris H. R., Mummery C. J., Mutamba J., Patterson M. C., Quiroz Y. T., Rabinovici G. D., Rommel A., Shulman M. B., Toledo-Sherman L. M., Weninger S., Wildsmith K. R., Worley S. L., Carrillo M. C. (2022). Current
directions in tau research: Highlights from Tau 2020. Alzheimers Dement..

[7] Rong J., Haider A., Jeppesen T. E., Josephson L., Liang S. H. (2023). Radiochemistry for positron emission
tomography. Nat. Commun..

[8] Deng X., Rong J., Wang L., Vasdev N., Zhang L., Josephson L., Liang S. H. (2019). Chemistry for Positron Emission Tomography:
Recent Advances in ^11^C-, ^18^F-, ^13^N-, and ^15^O-Labeling Reactions. Angew. Chem., Int. Ed. Engl..

[9] Krishnan H. S., Ma L., Vasdev N., Liang S. H. (2017). ^18^F-Labeling of Sensitive
Biomolecules for Positron Emission Tomography. Chemistry.

[10] Zhou X., Shi B., Huang G., Liu J., Wei W. (2024). Trends in cancer imaging. Trends Cancer.

[11] Wang, Y. ; Liao, W. ; Wang, L. ; Li, J. ; Huang, D. ; Cheng, W. ; Tian, J. ; Luan, P. Advance and Prospect of Positron Emission Tomography in Alzheimer’s disease research. Mol. Psychiatry 2025. 10.1038/s41380-025-03081-2. 40544200

[12] Fu H., Rong J., Chen Z., Zhou J., Collier T., Liang S. H. (2022). Positron Emission
Tomography (PET) Imaging Tracers
for Serotonin Receptors. J. Med. Chem..

[13] Chen Z., Haider A., Chen J., Xiao Z., Gobbi L., Honer M., Grether U., Arnold S. E., Josephson L., Liang S. H. (2021). The Repertoire of Small-Molecule PET Probes for Neuroinflammation
Imaging: Challenges and Opportunities beyond TSPO. J. Med. Chem..

[14] Hou L., Rong J., Haider A., Ogasawara D., Varlow C., Schafroth M. A., Mu L., Gan J., Xu H., Fowler C. J., Zhang M.-R., Vasdev N., Ametamey S., Cravatt B. F., Wang L., Liang S. H. (2021). Positron Emission
Tomography Imaging of the Endocannabinoid System: Opportunities and
Challenges in Radiotracer Development. J. Med.
Chem..

[15] Sun J., Xiao Z., Haider A., Gebhard C., Xu H., Luo H.-B., Zhang H.-T., Josephson L., Wang L., Liang S. H. (2021). Advances in Cyclic Nucleotide Phosphodiesterase-Targeted
PET Imaging and Drug Discovery. J. Med. Chem..

[16] Fu H., Chen Z., Josephson L., Li Z., Liang S. H. (2019). Positron
Emission Tomography (PET) Ligand Development for Ionotropic Glutamate
Receptors: Challenges and Opportunities for Radiotracer Targeting
N-Methyl-d-aspartate (NMDA), α-Amino-3-hydroxy-5-methyl-4-isoxazolepropionic
Acid (AMPA), and Kainate Receptors. J. Med.
Chem..

[17] Zhang J.-J., Fu H., Lin R., Zhou J., Haider A., Fang W., Elghazawy N. H., Rong J., Chen J., Li Y., Ran C., Collier T. L., Chen Z., Liang S. H. (2023). Imaging Cholinergic
Receptors in the Brain by Positron Emission Tomography. J. Med. Chem..

[18] Tissot C., Servaes S., Lussier F. Z., Ferrari-Souza J. P., Therriault J., Ferreira P. C. L., Bezgin G., Bellaver B., Leffa D. T., Mathotaarachchi S. S., Chamoun M., Stevenson J., Rahmouni N., Kang M. S., Pallen V., Margherita-Poltronetti N., Wang Y. T., Fernandez-Arias J., Benedet A. L., Zimmer E. R., Soucy J. P., Tudorascu D. L., Cohen A. D., Sharp M., Gauthier S., Massarweh G., Lopresti B., Klunk W. E., Baker S. L., Villemagne V. L., Rosa-Neto P., Pascoal T. A. (2023). The Association
of Age-Related and Off-Target Retention
with Longitudinal Quantification of [^18^F]­MK6240 Tau PET
in Target Regions. J. Nucl. Med..

[19] Blazhenets G., Soleimani-Meigooni D. N., Thomas W., Mundada N., Brendel M., Vento S., VandeVrede L., Heuer H. W., Ljubenkov P., Rojas J. C., Chen M. K., Amuiri A. N., Miller Z., Gorno-Tempini M. L., Miller B. L., Rosen H. J., Litvan I., Grossman M., Boeve B., Pantelyat A., Tartaglia M. C., Irwin D. J., Dickerson B. C., Baker S. L., Boxer A. L., Rabinovici G. D., La Joie R. (2023). [^18^F]­PI-2620 Binding Patterns
in Patients with Suspected Alzheimer Disease and Frontotemporal Lobar
Degeneration. J. Nucl. Med..

[20] Malpetti M., Kaalund S. S., Tsvetanov K. A., Rittman T., Briggs M., Allinson K. S. J., Passamonti L., Holland N., Jones P. S., Fryer T. D., Hong Y. T., Kouli A., Bevan-Jones W. R., Mak E., Savulich G., Spillantini M. G., Aigbirhio F. I., Williams-Gray C. H., O’Brien J. T., Rowe J. B. (2022). In Vivo ^18^F-Flortaucipir
PET Does Not Accurately Support the Staging of Progressive
Supranuclear Palsy. J. Nucl. Med..

[21] Schönecker S., Palleis C., Franzmeier N., Katzdobler S., Ferschmann C., Schuster S., Finze A., Scheifele M., Prix C., Fietzek U., Weidinger E., Nübling G., Vöglein J., Patt M., Barthel H., Sabri O., Danek A., Höglinger G. U., Brendel M., Levin J. (2023). Symptomatology in 4-repeat tauopathies
is associated with data-driven topology of [^18^F]-PI-2620
tau-PET signal. NeuroImage Clin..

[22] Brendel M., Barthel H., van Eimeren T., Marek K., Beyer L., Song M., Palleis C., Gehmeyr M., Fietzek U., Respondek G., Sauerbeck J., Nitschmann A., Zach C., Hammes J., Barbe M. T., Onur O., Jessen F., Saur D., Schroeter M. L., Rumpf J.-J., Rullmann M., Schildan A., Patt M., Neumaier B., Barret O., Madonia J., Russell D. S., Stephens A., Roeber S., Herms J., Bötzel K., Classen J., Bartenstein P., Villemagne V., Levin J., Höglinger G. U., Drzezga A., Seibyl J., Sabri O. (2020). Assessment of ^18^F-PI-2620 as a Biomarker in Progressive
Supranuclear Palsy. JAMA Neurol..

[23] Tezuka T., Takahata K., Seki M., Tabuchi H., Momota Y., Shiraiwa M., Suzuki N., Morimoto A., Nakahara T., Iwabuchi Y., Miura E., Yamamoto Y., Sano Y., Funaki K., Yamagata B., Ueda R., Yoshizaki T., Mashima K., Shibata M., Oyama M., Okada K., Kubota M., Okita H., Takao M., Jinzaki M., Nakahara J., Mimura M., Ito D. (2021). Evaluation of [^18^F]­PI-2620, a second-generation selective tau tracer, for
assessing four-repeat tauopathies. Brain Commun..

[24] Lindberg A., Tong J., Zheng C., Mueller A., Kroth H., Stephens A., Mathis C. A., Vasdev N. (2025). Radiosynthesis, In
Vitro Characterization, and In Vivo PET Neuroimaging of [^18^F]­F-4 for Tau Protein: A First-in-Human PET Study. ACS Chem. Neurosci..

[25] Li L., Liu F. T., Li M., Lu J. Y., Sun Y. M., Liang X., Bao W., Chen Q. S., Li X. Y., Zhou X. Y., Guan Y., Wu J. J., Yen T. C., Jang M. K., Luo J. F., Wang J., Zuo C., Progressive Supranuclear
Palsy Neuroimage, I. (2021). Clinical
Utility of ^18^F-APN-1607 Tau PET Imaging in Patients with
Progressive Supranuclear
Palsy. Mov. Disord..

[26] Lindberg A., Knight A. C., Sohn D., Rakos L., Tong J., Radelet A., Mason N. S., Stehouwer J. S., Lopresti B. J., Klunk W. E., Sandell J., Sandberg A., Hammarström P., Svensson S., Mathis C. A., Vasdev N. (2021). Radiosynthesis,
In Vitro and In Vivo Evaluation of [^18^F]­CBD-2115 as a First-in-Class
Radiotracer for Imaging 4R-Tauopathies. ACS
Chem. Neurosci..

[27] Graham T. J. A., Lindberg A., Tong J., Stehouwer J. S., Vasdev N., Mach R. H., Mathis C. A. (2023). In Silico Discovery
and Subsequent Characterization of Potent 4R-Tauopathy Positron Emission
Tomography Radiotracers. J. Med. Chem..

[28] Stehouwer J. S., Huang G., Saturnino Guarino D., Debnath M. L., Polu A., Geib S. J., Lopresti B., Ikonomovic M. D., Mason N., Mach R. H., Mathis C. A. (2025). Structure-Activity
Relationships and Evaluation of 2-(Heteroaryl-cycloalkyl)-1H-indoles
as Tauopathy Positron Emission Tomography Radiotracers. J. Med. Chem..

[29] Lindberg A., Murrell E., Tong J., Mason N. S., Sohn D., Sandell J., Strom P., Stehouwer J. S., Lopresti B. J., Viklund J., Svensson S., Mathis C. A., Vasdev N. (2024). Ligand-based design of [^18^F]­OXD-2314 for
PET imaging in non-Alzheimer’s disease tauopathies. Nat. Commun..

[30] Murrell E., Narciso L., Desmond K. L., Chow C., Lindberg A., Garcia A., Mathis C. A., Svensson S., Strafella A. P., Vasdev N. (2025). First-in-human PET neuroimaging of
[^18^F]­OXD-2314. Eur. J. Nucl. Med.
Mol. Imaging.

